# Anterior Quadratus Lumborum block area comparison in the three different volumes of Ropivacaine: a double-blind, randomized controlled trial in healthy volunteers

**DOI:** 10.1186/s12871-022-01922-z

**Published:** 2022-11-29

**Authors:** Liang Shao, Xu Luo, Yingchao Ye, Le Liu, Yaoyao Cai, Yun Xia, Thomas J. Papadimos, Quanguang Wang, Linmin Pan

**Affiliations:** 1 Department of Anesthesiology, the People’s Hospital of Yuhuan, Taizhou City, 317600 Zhejiang Province China; 2 Department of Anesthesiology, the First Affiliated Hospital, Wenzhou Medical University, Shangcai village, Nanbaixiang town, Ouhai District, Wenzhou City, 325000 Zhejiang Province China; 3 Department of Burn, the First Affiliated Hospital, Wenzhou Medical University, Shangcai village, Nanbaixiang town, Ouhai District, Wenzhou City, 325000 Zhejiang Province China; 4 Department of Anesthesiology, Ohio State University Wexner Medical Center, Columbus, OH USA

**Keywords:** Anesthesia, conduction, Ultrasound, Volunteers

## Abstract

**Background:**

In abdominal surgery, ultrasound-guided anterior quadratus lumborum blocks (QLB) are performed to induce analgesia. However, no study reported suitable volumes of the anterior QLB for the different postoperative analgesia regions. Therefore, this prospective randomized controlled study assessed the dermatomal spread and analgesic effects of the three different volumes of a local anesthetic for anterior QLB.

**Methods:**

Ultrasound-guided anterior QLB was performed at the L2 level on 30 healthy volunteers. The volunteers were randomized to receive 20 ml (*n* = 10), 30 ml (n = 10), and 40 mL (n = 10) of 0.375% ropivacaine. The cutaneous sensory blocked area (CSBA), the number of block dermatomes, and the block duration time were measured by determining the extent of the cold sensation.

**Results:**

The CSBA was significantly larger in the 40 ml group than in the 30 (*P* = 0.001; 1350.6 ± 234.4 vs. 1009.5 ± 151.6 cm^2^) and 20 ml groups (*P* < 0.001; 1350.6 ± 234.4 vs. 808.1 ± 120.5 cm^2^). Similarly, the number of blocked dermatomes was significantly higher in the 40 ml group than in the 30- and 20-ml groups. However, no significant difference was observed in block duration among the groups.

**Conclusions:**

No difference was observed in block duration with the various volumes of 0.375% ropivacaine. However, the larger volume for anterior QLB contributed to a larger area of cutaneous sensory blockade. Appropriate volumes in anterior QLB can create suitable postoperative analgesia levels for the different operative sites.

**Trial registration:**

The study was registered in the Chinese Clinical Trial Registration Center on www.chictr.org.cn on 27th April 2018 (registration number: ChiCTR-IOR-17010853).

**Supplementary Information:**

The online version contains supplementary material available at 10.1186/s12871-022-01922-z.

## Introduction

Ultrasound-guided quadratus lumborum block (QLB) is a fascial plane block technique proposed and optimized by Blanco et al. [1]. It is primarily used for perioperative analgesia during abdominal surgery. The local anesthetic diffusion into the thoracolumbar fascia relieves somatic and visceral pain [2].

No consensus exists on the optimal QLB local anesthetic volume. The QLB can result in diverse sensory suppression via a wide distribution of local anesthetics in different doses [3, 4]. Investigators have injected dye volumes of 20 [5], 30 [6], and 40 ml [7] in the anterior QLB on cadavers during the performance of the anterior QLB and found variations in the blockade extent. However, no randomized controlled studies have been reported on the effect of different volumes of local anesthetics on the extent of anterior QLB.

Therefore, we investigated the effects of different volumes of local anesthetic on the range and duration of cold sensation disappearance in the ultrasound-guided anterior QLBs in volunteers. This study was performed on healthy volunteers to assess the effectiveness of 20 ml vs. 30 ml vs. 40 ml of 0.375% ropivacaine in ultrasound-guided QLB. The primary outcome of the study was the extent of the cutaneous sensory blockade area (CSBA). The secondary outcomes included (1) the number of blocked dermatomes and (2) the blockade duration.

## Methods

### Ethics approval

This was a prospective, double-blind, randomized controlled study. This study was approved by the institutional review board of the First Affiliated Hospital of Wenzhou Medical University (Chairperson Professor Rong Jin), Ouhai District, Wenzhou, Zhejiang, China, on 13/03/2017. The study was registered in the Chinese Clinical Trial Registration Center on www.chictr.org.cn (registration number: ChiCTR-IOR-17010853). Written informed consent was obtained from all volunteers before the study procedures.

### Selection and description of volunteers

Adult volunteers (18–45 years old, BMI:18-30 kg/m^2^) with ASA-I or II scheduled for ultrasound-guided QLB were recruited between February 2019 and October 2020.

Exclusion criteria: Communication disorders, comprehension disorders, psychological disorders, history of local anesthetic or drug allergy, dermatological disorders, abnormal heart and/or lung function, history of acute or chronic low back pain, current infectious disease, and abnormal blood platelet or coagulation function. Written informed consent was obtained from all volunteers.

Patients were randomized and were equally distributed into three groups, viz. 20 ml, 30 ml, and 40 ml group, using a random number generator by the two assistants, who were not involved in other parts of this trial. The volunteers and anesthesiologists who assessed the sensory block area were kept blind for the participant’s group assignment.

### Block procedure

**Fig. 1:**
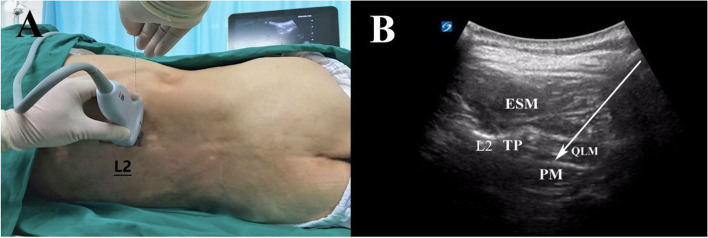
Photographs and ultrasonographic images demonstrate the lateral decubitus position for the QLB. **A** Depiction of needle position and probe placement in each group; **B** corresponding ultrasonographic images. Arrow indicates the needle trajectory orientation. ESM: erector spine muscle; TP: transverse process; PM: psoas muscle; QLM: quadratus lumborum

The standard monitors were attached to the volunteers in the operating room, where noninvasive blood pressure, electrocardiogram, and pulse oximetry were attached for continuous vital signs monitoring. Secured venous access was established in the contralateral upper limb. The mean blood pressure and heart rate were recorded over 30 seconds every 5 minutes at baseline till 60 minutes after block procedures. The volunteers were placed in the left decubitus position; the sacrum and the L5 spinous process were identified using a 2-5 MHz low-frequency curve ultrasound probe (SonoSite X-Porte, SonoSite Inc., Bothell, WA, USA) placed along the posterior midline. Next, the L2 transverse process, the psoas major, and the erector spinae muscle were identified in the L2 spinous process by sliding the probe cephalad. After subcutaneous anesthesia with 1 mL of 1% lidocaine, the QLB was performed under ultrasound guidance with a 100 mm 22-gauge echogenic needle (22G, KDL, Wenzhou, China) in-plane technique. The needle tip was advanced to the space between the quadratus lumborum and the psoas major muscle. After confirming that air and blood were not aspirated, 0.375% ropivacaine was injected in either 20, 30, or 40 mL, according to the group assignment (Fig. 1).

### Measurement and calculation

Sixty minutes after the local anesthetic injection, the number of blocked dermatomes was assessed in the craniocaudal direction along the midaxillary line. A cold test was performed with ice to evaluate the blocked area. The ice cube was moved radially at a speed of 2 cm/s to find the extent of the sensory changes. The ventral movement was performed from the left midclavicular line to the anterior midline and then to the right midaxillary line. The dorsal movement was performed from the left midclavicular line to the posterior midline and then to the right mid-axillary line. The sensation was evaluated on a 3-point scale: 2 = skin feels normal, 1 = significantly reduced cold sensation, 0 = disappearance of cold sensation [8]. The changes in sensation were marked from 2 to 1. The marks were further connected with a solid line to construct a distribution map of the dermal anesthesia (If any position was without changing 2 to 1, we marked the site 2 to 0 instead). Next, we connected the red dots to map the cutaneous sensory block areas (images were obtained). We covered the cutaneous sensory block areas with a 60*80 cm transparent rectangular film and transferred all the surface landmarks/lines onto it. The highest and lowest blocked segments (as delineated by evaluation of the areas of cold sensation changes in volunteers following 1 h of the blockade) were measured and recorded. Block duration tests were performed every hour from the onset of the sensory block until complete remission. Two weeks after the intervention, the primary investigator had a follow-up phone interview with the volunteers to ascertain any complications related to the trial intervention.

### Statistics analysis

Sample size calculation was performed using the Power Sample Size Program (PASS.11.0). Based on the pilot study, the mean cutaneous areas of sensory blockade 60 minutes after QLB were 810 cm^2^ (SD, 125) in the 20 ml group, 1032 cm^2^ (SD, 141) in the 30 ml group, and 1355 cm^2^ (SD, 134) in 40 ml group. We accepted a two-tailed significance level of significance at 0.05 and a power of 0.9 (α = 0.05 and β = 0.1). We determined that the 9 volunteers per group were the required minimum sample size. Considering the possible dropouts, the final sample size was 10 volunteers per group.

Statistical analysis was performed with SPSS 21.0 software. The Shapiro-Wilk test was used for testing the normal distribution. The measured data of normal distribution were expressed as the mean ± SD. The measured data of non-normal distribution were expressed as the median (quartile). Type data were expressed as the frequency. One-way analysis of variance was used for age, height, weight, and cutaneous sensory block area. The Bonferroni test was used for the pairwise comparison. Gender and ASA classification were analyzed by Fisher’s exact test. Block segments were analyzed by the Kruskal-Wallis H test. *P* < 0.05 was regarded as statistically significant. Kaplan-Meier survival curves were constructed to examine the block duration difference between the groups.

## Results

A total of 35 volunteers were enrolled in the study. Five volunteers were excluded, and the spread of the local anesthetic solution during QLB was confirmed at appropriate locations in all enrolled volunteers.

No significant differences were observed in regard to age, height, weight, BMI, and gender (*P* > 0.05, Table 1).

**Table 1:** Participants Characteristics

Variable	20 mL (***n*** = 10)	30 mL (n = 10)	40 mL (n = 10)	***P***-value
Age, y	37.9 ± 7.7	39.1 ± 7.9	38.6 ± 6	0.933
Height, cm	168.4 ± 5.5	168.9 ± 4.7	167.5 ± 6.0	0.844
Weight, kg	64.7 ± 6.0	68.9 ± 6.7	65.3 ± 7.6	0.344
BMI, kg/cm2	22.8 ± 1.99	24.1 ± 1.6	23.2 ± 2.0	0.312
Gender, F/M	1/9	1/9	4/6	0.301
ASA, I/II	9/1	9/1	9/1	1

Data are expressed as means ± SD or as absolute numbers

**Fig. 2:**
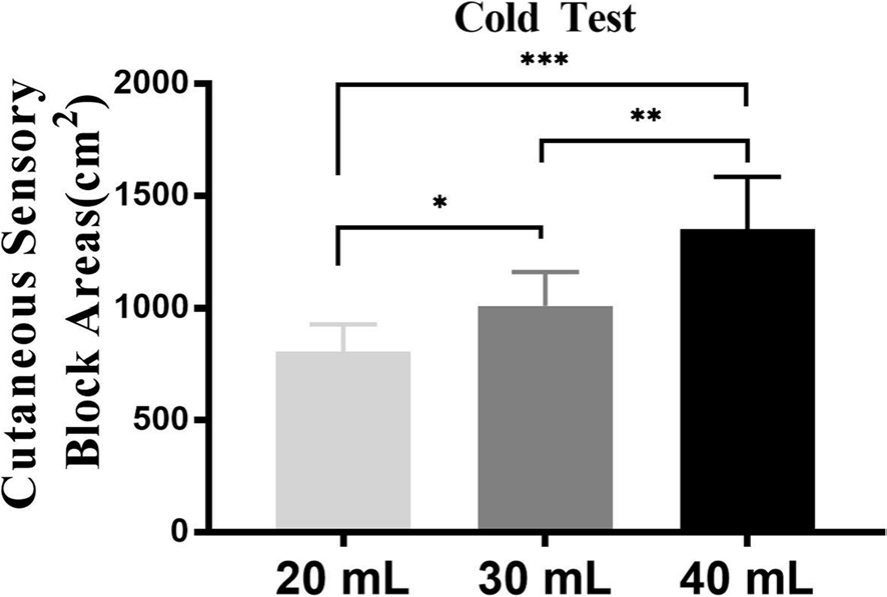
Sensory block distribution at 1 hour after the block. The solid red line constructed a distribution map of the dermal anesthesia (the cold sensation was significantly reduced or disappeared)

**Fig. 3:**
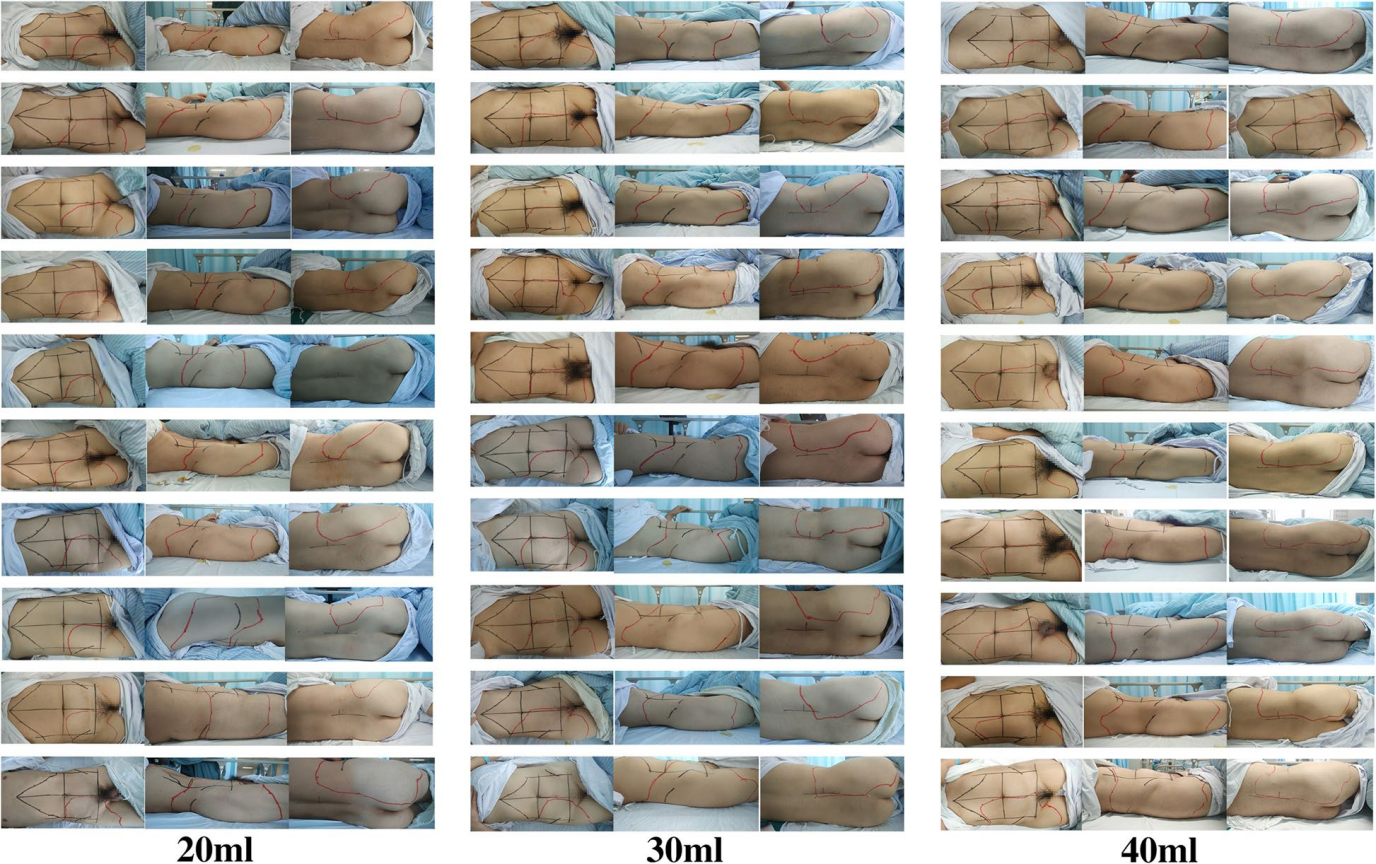
The cutaneous sensory block areas are expressed as mean ± SD. After the cold test, significant differences were observed among the 20-, 30-, and 40-mL groups. Data is expressed as mean ± SD; *p < 0.05, **p < 0.01, ***p < 0.001

All the volunteers showed cutaneous sensory loss on the blocked side 60 minutes after the QLB (Fig. 2). The cutaneous sensory block areas (CSBA) assessed by the cold test (Fig. 3) were significantly larger in the 40 mL of 0.375% ropivacaine group than in the 30 (*P* = 0.001;1350.6 ± 234.4 vs. 1009.5 ± 151.6 cm^2^) and 20 ml groups (*P* < 0.001; 1350.6 ± 234.4 vs. 808.1 ± 120.5 cm^2^). In addition, the CSBA in the 30 mL group was significantly larger than the 20 mL group (*P* = 0.049; 1009.5 ± 151.6 vs. 808.1 ± 120.5 cm^2^).

**Fig. 4:**
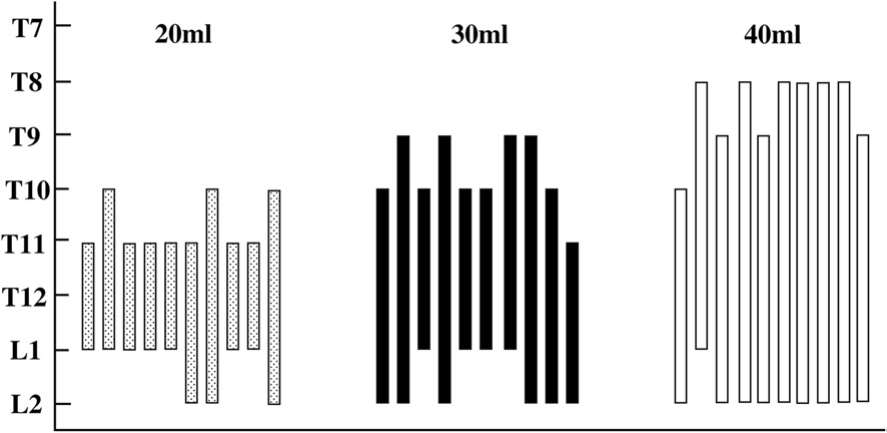
Dermatome effects after quadratus lumbar muscle block for each participant receiving 20, 30, or 40 mL of 0.375% ropivacaine, respectively

**Fig. 5:**
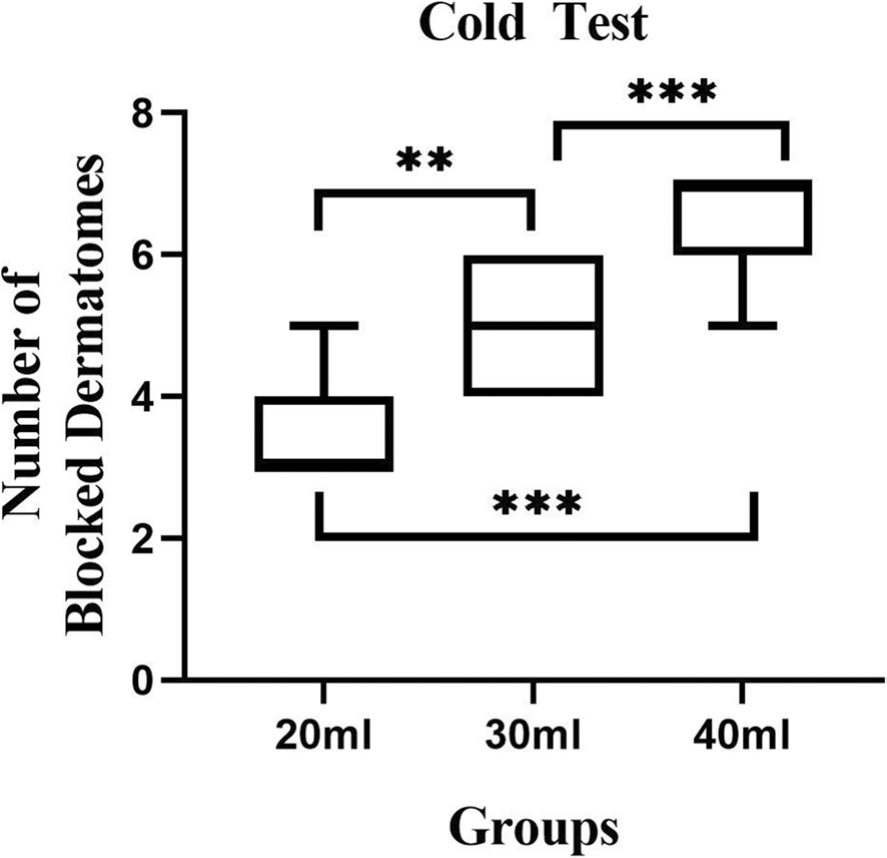
The number of blocked dermatomes in the 20, 30, and 40 ml groups. There are significant differences in the number of blocked dermatomes among the 20-, 30-, and 40-mL groups. Data is expressed as median (quartile); **p < 0.01, ***p < 0.001

Most of the cephalad-affected dermatomes assessed by the cold test were observed at T10 in the 20 ml, T9 in the 30 ml, and T8 in the 40 ml group (Fig. 4). The caudal dermatomes reached L2 in all the groups. The number of affected dermatomes was significantly larger for 40 mL of 0.375% ropivacaine than 30 mL (*P* = 0.001; median [interquartile range], 7 [6 to 7] vs. 5 [4 to 6] dermatomes) and 20 ml groups (*P* < 0.001; median [interquartile range], 7 [6 to 7] vs. 3 [3 to 4] dermatomes). Dermatomal distribution in the volunteers receiving 30 mL was significantly larger than 20 mL of 0.375% ropivacaine (*P* = 0.002; median [interquartile range], 5 [4 to 6] vs. 3 [3 to 4] dermatomes) (Fig. 5).

**Fig. 6:**
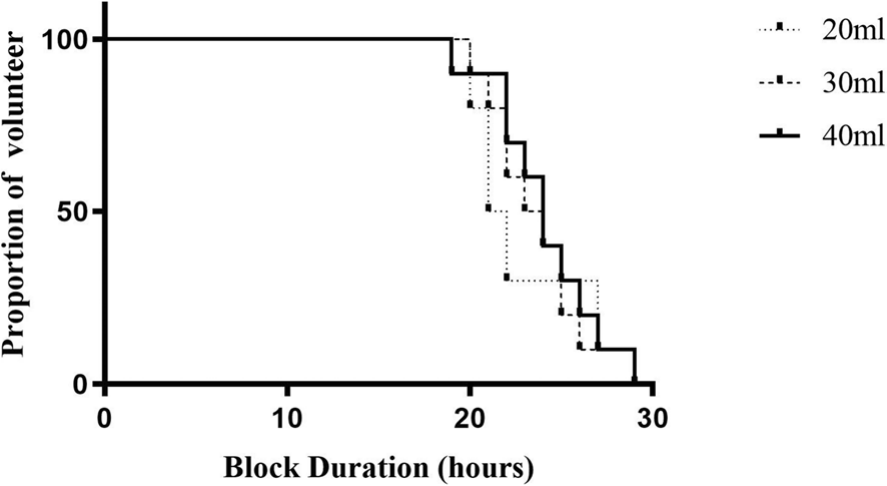
Survival table for the block duration. No significant difference was observed among the 20-, 30-, and 40-ml groups (median time, 21 vs. 24 vs. 24 h, *P* = 0.712)

No significant difference was observed in the cold sensation block duration among the 20, 30, and 40 ml groups (median time, 21 vs. 24 vs. 24 h, *P* = 0.712) (Fig. 6).

There was no evidence of hemodynamic deterioration during the procedure or at 60 minutes post-block (Hemodynamic deterioration was defined as the decline of systolic blood pressure or heart rate by 25% or greater from the baseline). Moreover, no clinical signs of local anesthetic toxicity were observed at any dose. No complications were recorded during the follow-up interviews.

## Discussion

This study demonstrated that the ultrasound-guided anterior QLB could produce a wider area of the blockade and an increased number of blocked dermatomes with the increased volumes of 0.375% ropivacaine. The most cephalad-affected dermatomes assessed by the cold test were at T10, T9, and T8 with the injection of 20, 30, and 40 ml ropivacaine, respectively. The variable injection volume of 0.375% ropivacaine might be suitable for different kinds of surgery.

Previous studies have reported several ultrasound-guided anterior QLB using in-plane methods, including L1-L2 level [9], L2 level [10], L3 level [11], and L4 level [12]. Previous studies have confirmed that the L2 level produced a widespread cutaneous sensory blockade compared with the L4 level [10]. In addition, many other studies have located the midpoint of the costal arch and iliac crest (consistent with the L2 level normally) for the QLB [13]. Therefore, we decided to perform the anterior QLB at the L2 level, which also increased the stability of our operation with a better cephalad spread of local anesthetics.

The anterior quadratus lumborum block injection volume used in the study may have contributed to an increased spread and broader coverage at the lower to mid-thoracic region [9]. The diffusion of local anesthetics in the thoracolumbar fascia is the primary mechanism of the QLB [14]. Furthermore, we demonstrated that the number of thoracic dermatomes without cold sensation increases with the increase in the volume of local anesthetic. Moreover, our results concur with the previously reported anatomical and clinical literature. Dam et al. [15] and Elsharkawy et al. [16] reported the possibility of thoracic paravertebral space spread of fresh cadavers in anterior QLBs with a 30 ml volume. In our study, higher volume regimes increased the range of our blocks and reached a higher thoracic dermatomal distribution. Clinically, we chose different injection levels (L1–2, L3, L4) of QLB for different kinds of surgery (e.g., nephrectomy [17], cesarean delivery [18], hip arthroplasty [19]) to ease the spread of local anesthetic for fulfilling the requirement of local analgesia. Our study shows that different volumes can be used at one injection level at L2 to acquire a different block area for different clinical applications.

QLB provides a new method of trunk nerve block, currently used for postoperative analgesia for abdominal surgery. Consistent with our results for the duration time of QLB, Nasir et al. summarized that using a single injection of QLB for postoperative analgesia in cesarean delivery had an effective analgesia time for up to 24 hours [20]. However, uncertainty exists in contrast to peripheral nerve blocks about the relative local anesthetic volume and block duration for fascial plane blocks [21]. As previously demonstrated, a higher volume was not associated with a longer duration of sensory blockade in serratus plane block [22]. To the best of our knowledge, no evidence was reported for the plausible relationship between the volume of local anesthetic and the effective duration of a QLB. As QLB is a fascial plane block, high-volume regional anesthesia would result in the broad spread of local anesthetic in the interfascial plane, but not with a longer effective time. Ropivacaine 0.375% is a commonly selected concentration in QLB for postoperative analgesia in recent studies [23], and thus serves as a reference concentration.

Christian et al. injected 30 mL of 0.375% ropivacaine bilaterally (60 mL in total) in the fascial interspace of quadratus lumborum and psoas major muscle without any local anesthetic toxicity symptoms [24]. Takeshi et al. suggested that QLB with 150 mg of ropivacaine was safe, and the plasma ropivacaine concentration was below the neurotoxic level [4]. Our study showed that the unilateral 150 mg ropivacaine was safe in anterior QLB. However, as the larger volumes of ropivacaine contributed to a larger area of cutaneous sensory blockade, the safety of bilateral 30 or 40 ml 0.375% ropivacaine still needs to be validated.

Our study had some limitations: (1) the study only examined the relationship between volume and cold sensation, which cannot be extrapolated to the postsurgical pain, (2) The documented time points of all data had a 1 h lag time behind the nerve block; thus, these time points could not be defined as the “peak period” of block effect in any of the three-volume regimens, (3) we only studied the pharmacodynamics, not pharmacokinetics of 0.375% ropivacaine.

## Conclusions

We studied the changes in the cutaneous sensory loss areas and the block segments after 3 different injection volumes (20, 30, or 40 mL) of 0.375% ropivacaine in the anterior quadratus lumbar muscle block. The cutaneous sensory block areas and the number of blocked dermatomes increased significantly with the increase in local anesthetic volume. In addition, affected dermatomes of 0.375% of ropivacaine in 20, 30, and 40 ml groups reached their highest levels at T10, T9, and T8, respectively, with the lowest level at L2. In summary, the larger volumes used for anterior QLB contributed to a larger area of the cutaneous sensory blockade with a more extensive dermatomal distribution. Therefore, our approach for using different volumes in fascial plane blocks might help create a more suitable and satisfactory postoperative analgesia for the different operative sites.

## Supplementary Information

**Additional file 1.**

## Data Availability

The datasets used and/or analyzed during the current study are available from the corresponding author upon reasonable request.

## Abbreviations

QLB: quadratus lumborum blocks
CSBA: cutaneous sensory blocked area
BMI: Body mass index
