# Supplementary material for: Anterior Quadratus Lumborum block area comparison in the three different volumes of Ropivacaine: a double-blind, randomized controlled trial in healthy volunteers
Source: BMC Anesthesiol. 2022 Nov 29;22:365. doi: 10.1186/s12871-022-01922-z (PMC9706850; doi:10.1186/s12871-022-01922-z)
Supplement: Supplementary file 1 — Additional file 1. [file 12871_2022_1922_MOESM1_ESM.doc]

**
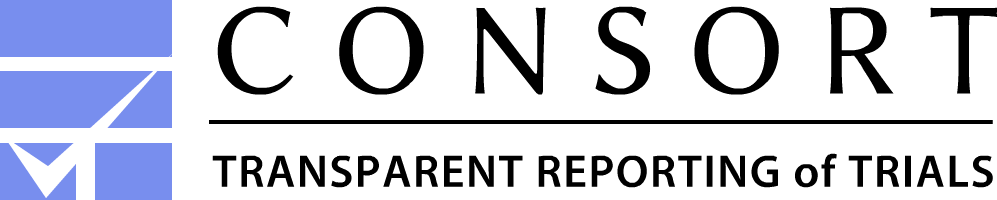
**

**CONSORT 2010 Flow Diagram**

**Allocation**

**Analysis**

**Enrollment**

Assessed for eligibility (n= 35)

Excluded (n= 5)

♦  Not meeting inclusion criteria (n=3)

♦  Declined to participate (n= 2)

♦  Other reasons (n= 0)

**Group 20ml**

Allocated to intervention (n=10)

Randomized (n=30)

**Analysed (n=10)**

**Analysed (n=10)**

**Analysed (n=10)**

**Group 30ml**

Allocated to intervention (n=10)

**Group 40ml**

Allocated to intervention (n=10)
